# Natural Variation in Stomatal Responses to Environmental Changes among *Arabidopsis thaliana* Ecotypes

**DOI:** 10.1371/journal.pone.0117449

**Published:** 2015-02-23

**Authors:** Sho Takahashi, Keina Monda, Juntaro Negi, Fumitaka Konishi, Shinobu Ishikawa, Mimi Hashimoto-Sugimoto, Nobuharu Goto, Koh Iba

**Affiliations:** 1 Department of Biology, Faculty of Sciences, Kyushu University, Fukuoka, Japan; 2 RIKEN BioResource Center, Koyadai, Tsukuba, Ibaraki, Japan; National Taiwan University, TAIWAN

## Abstract

Stomata are small pores surrounded by guard cells that regulate gas exchange between plants and the atmosphere. Guard cells integrate multiple environmental signals and control the aperture width to ensure appropriate stomatal function for plant survival. Leaf temperature can be used as an indirect indicator of stomatal conductance to environmental signals. In this study, leaf thermal imaging of 374 *Arabidopsis* ecotypes was performed to assess their stomatal responses to changes in environmental CO2 concentrations. We identified three ecotypes, Köln (Kl-4), Gabelstein (Ga-0), and Chisdra (Chi-1), that have particularly low responsiveness to changes in CO2 concentrations. We next investigated stomatal responses to other environmental signals in these selected ecotypes, with Col-0 as the reference. The stomatal responses to light were also reduced in the three selected ecotypes when compared with Col-0. In contrast, their stomatal responses to changes in humidity were similar to those of Col-0. Of note, the responses to abscisic acid, a plant hormone involved in the adaptation of plants to reduced water availability, were not entirely consistent with the responses to humidity. This study demonstrates that the stomatal responses to CO2 and light share closely associated signaling mechanisms that are not generally correlated with humidity signaling pathways in these ecotypes. The results might reflect differences between ecotypes in intrinsic response mechanisms to environmental signals.

## Introduction

Plants have evolved the ability to adapt to environmental signals in order to optimize plant growth under various conditions. Plants sense changes in their natural environments, and alter their development and physiology in response to these changes. Guard cells play a key role in responding to environmental changes [1]. Guard cells regulate stomatal apertures by integrating environmental signals and endogenous hormone stimuli. Therefore, guard cells have been studied extensively as a model system for dissecting the dynamics and mechanisms of environment sensing [2]. Genetic studies of mutant varieties promote our understanding of guard cell responses in plants [3, 4]. This approach usually focuses on one gene at a time, however, the signaling pathways controlling these responses are likely to be integrated into complex networks rather than single independent pathways [5].

The model plant *Arabidopsis thaliana* (L.) Heynh is widely spread geographically. It has been used to study the genetic and molecular bases of complex traits focused on natural genetic and phenotypic variations [6]. Such studies in wild species can provide information about the molecular changes related to plant adaptation in diverse natural environments [7]. However, thus far there have been very few studies focusing on the diversity of stomatal responses to environmental changes in phenotypically divergent ecotypes [8]. On the other hand, extensive studies using *Arabidopsis* mutants have shed light on the molecular mechanisms controlling guard cell responses to environmental stimuli [9–16]. For example, CO_2_-insensitive mutants were isolated using leaf thermal imaging, and these studies identified important components of pathways that regulate stomatal aperture. These components include HT1 protein kinase, a key molecular regulator of high CO_2_-induced stomatal closure [12], SLAC1, an S-type anion channel [14], and PATROL1, which plays a role in tethering H^+^-ATPase to the plasma membrane during stomatal opening [16]. Thus we expect that studying stomatal responses to environmental signals in a wide variety of *Arabidopsis* ecotypes will contribute to our understanding of these complex response mechanisms.

Stomatal pores serve as major gateways for both CO_2_ influx into plants from the atmosphere and transpirational water loss from plants. Transpiration causes leaf cooling because evaporation of water is associated with heat loss. Leaf surface temperature can be measured continuously and non-destructively using infrared thermography, and this provides a convenient indicator of the transpiration of individual plants [4, 17, 18]. In this study, we measured leaf temperature changes that occurred in response to changes in CO_2_ concentrations in 374 *Arabidopsis* ecotypes. We identified three ecotypes, Kl-4, Ga-0 and Chi-1, with particularly low responsiveness to CO_2_ and used these in further studies. We compared the three ecotypes with the commonly used ecotype Col-0, and measured their stomatal responses to CO_2_, light and dark, high and low humidity and abscisic acid (ABA). Our data revealed that the ecotypes with low CO_2_ responsiveness are impaired in their responses to both high CO_2_ concentrations and light, but they showed normal stomatal responses to humidity. This suggests that the signaling pathway controlling stomatal responses to CO_2_ and light is different from the pathway controlling responses to changes in humidity.

## Materials and Methods

### Plant materials

Wild-type *Arabidopsis thaliana* (L.) Heynh ecotypes (374 ecotypes) were obtained from the Arabidopsis Biological Resource Center (Ohio State University) (S1 Table). Plants were grown on solid MS medium for 18 days in a growth chamber [constant white light of 80 μmol m^-2^ s^-1^ at 22°C and 40% relative humidity (RH)], and then transplanted into vermiculite pots with Hyponex 6-10-5 fertilizer, diluted 1,000-fold (Hyponex, Osaka, Japan). Plants at 21–24 days old were used for leaf temperature measurements and plants at 24–27 days old were used for all other experiments.

### Thermal imaging

Plants grown in a growth chamber were transferred to a custom-made growth cabinet (constant white light of 100 μmol m^-2^ s^-1^ at 22°C, 40% RH) equipped with an automatic CO_2_ control unit (TMC-LW1208A/K, TM Systems Ltd.,). Thermal images were captured under different CO_2_ concentrations using a thermography camera (Thermography R300, NEC/AVIO) and an InfRec Analyzer NS9500 Standard (NEC Avio Infrared Technologies CO. Ltd).

### Stomatal conductance measurement

The whole plant stomatal conductance to water vapor (*g*_*s*_) was measured using a portable gas exchange system (GFS-3000, Heinz Walz) equipped with a 3010-A *Arabidopsis* chamber. The temperature (22°C), flow rate (750 μmol s^-1^), light intensity (150 μmol m^-2^ s^-1^), and RH (40%) were kept constant throughout the gas exchange experiments. The *g*_*s*_ response to CO_2_ was measured by increasing CO_2_ concentrations every 1 h in five steps (0, 200, 400, 600 and 1,000 ppm) before plants were incubated under 0 ppm CO_2_ concentration for 2 h.

### Stomatal size, density, and index measurements and aperture response analyses

Stomatal densities, index, guard cell lengths, and stomatal apertures were measured using photographs of epidermal peels taken with a digital camera attached to a microscope (IX71, Olympus). Guard cell lengths were used as a measure of stomatal size. Abaxial epidermal peels from two leaves of each of three plants were used for measurements of stomatal density, stomatal index and guard cell length. Sixty randomly chosen test areas of 0.14 mm^2^ from the six leaves per ecotype were analyzed. Stomatal index was defined as the number of stomata per total number of epidermal cells. For the aperture response analyses, three plants per ecotype were incubated under various CO_2_ concentrations, light intensity, or humidity conditions. Abaxial epidermal peels were then taken from two leaves of each plant, and these were used for aperture measurements within 15 min after peeling. Plants used for CO_2_ response measurements were incubated under a high CO_2_ concentration of 700 ppm for 1 h after incubation in CO_2_ concentration of 0 ppm for 2 h. Plants used for light response measurements were incubated under dark conditions for 2 h, then illuminated with white light at an intensity of 250 μmol m^-2^ s^-1^ for 3 h. Plants used for humidity response analyses were incubated under low humidity (40% RH) for 1 h after incubation under high humidity (80% RH) for 3 h. Stomatal aperture measurements were also performed using ABA-treated epidermal peels as described previously [19], with minor modifications. The epidermal peels were floated on a medium containing 30 mM KCl, 5 mM Mes-KOH (pH 6.15) and 1 mM CaCl_2_ and incubated in a growth chamber under light for 1 h. ABA from a stock solution in dimethyl sulfoxide was added to the same medium with a final concentration of 2 μM. The peels were then transferred to fresh medium with or without ABA and incubated for a further 2 h before stomatal apertures were measured. Statistical analyses were performed using the Student's t test.

### Isolation of guard cell protoplasts (GCPs)

GCPs were isolated as described previously [20], with modifications described [21].

### Measurement of organic and inorganic ions in GCPs

The GCPs (3 × 10^6^ cells) were suspended in 0.4 M mannitol, 10 mM KCl, 0.5 mM CaCl_2_, 0.5 mM MgCl_2_ and 5 mM MES-Tris buffer (pH 6.0) and illuminated with white light (80 μmol m^-2^ s^-1^ for 1 h) as described elsewhere [22] before isolation of the ions. The GCPs were ground into a fine powder, suspended in distilled water and boiled for 5 min. The suspensions were applied to Microcon YM-10 centrifugal filters (Millipore), and the organic/inorganic ions were measured in the filtrate.

The measurement of organic and inorganic ions was performed on an HPLC (Prominence, Shimadzu). The chromatographic system consisted of a Shimadzu Model system controller (SLC-10AVP), conductivity detector (CDD-10ADVP), and column oven (CTO-20A). K^-^ and Na^+^ were separated using a LC-10ADVP pump, a column Shim-pack IC-C4 (150 mm × 4.6 mm) and a guard column Shim-pack IC-GC4 (10 mm × 4.6 mm) with a mobile phase (3.2 mM Bis-Tris, 8 mM p-hydroxybenzoic acid, 50 mM Boric acid) and a flow rate of 1.0 ml min^-1^ at 40°C. Cl^-^ and Malate^2-^ were separated with a LC-20AD pump, a column Shim-pack IC-A3 (150 mm × 4.6 mm) and guard column Shim-pack IC-GA3 (10 mm × 4.6 mm) with a mobile phase (3/4 dilution of 3.2 mM Bis-Tris, 8 mM p-hydroxybenzoic acid, 50 mM boric acid) and a flow rate of 1.2 ml min^-1^ at 40°C. Statistical analyses were performed using the Student's t test.

## Results

### Comprehensive investigation of CO_2_ responses in *Arabidopsis* ecotypes

**Fig 1:**
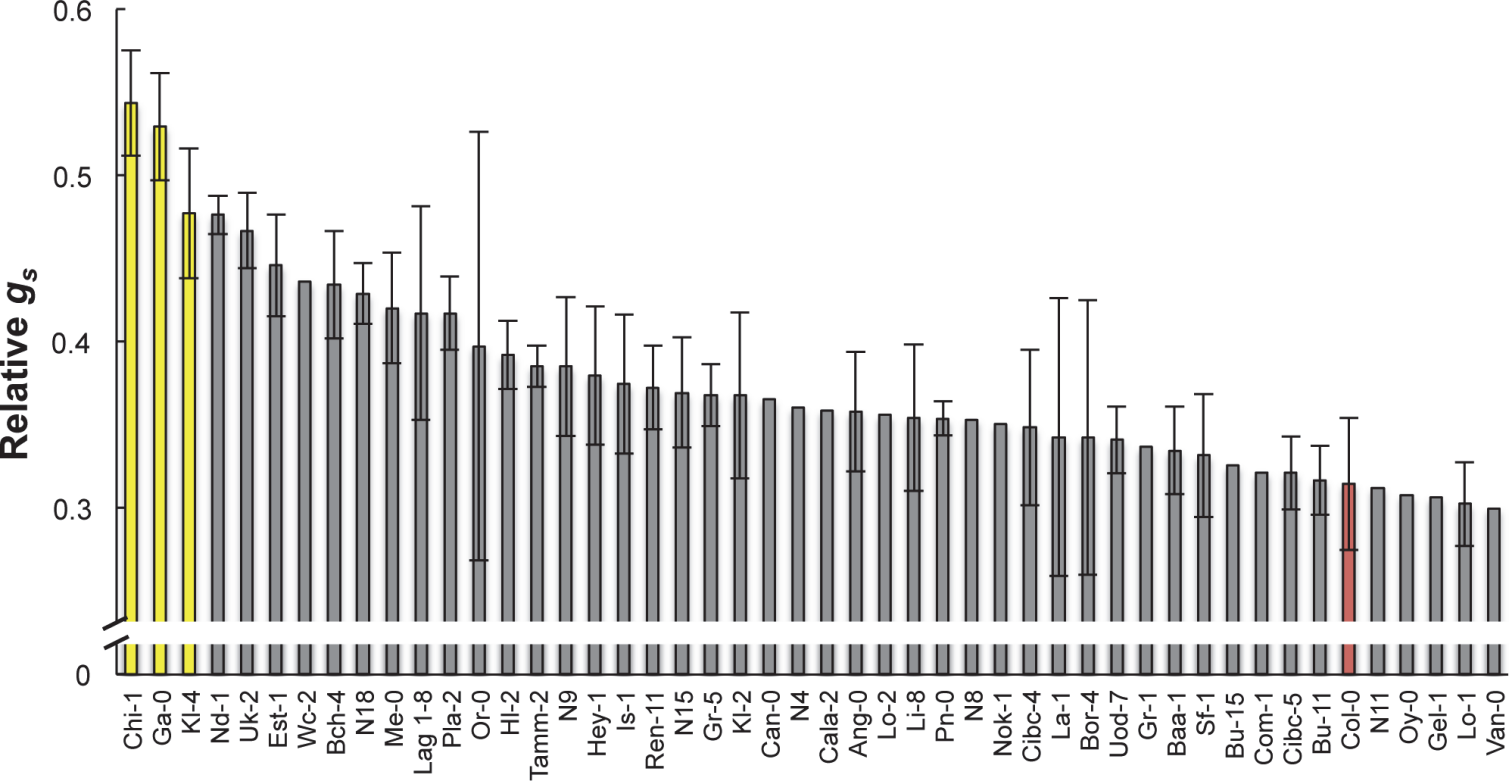
Stomatal conductance in *Arabidopsis* ecotypes that have demonstrated a low CO_2_ responsiveness. The plants were exposed to 0 ppm CO_2_ for 2 h and then transferred to 1,000 ppm CO_2_ for 1.5 h at 40% RH. Relative conductance levels (Relative *g*_*s*_) were calculated as (*g*_*s*_ at 1,000 ppm CO_2_)/(*g*_*s*_ at 0 ppm CO_2_); large values represent small responses. Data presented are means ± SE (*n* = 3). The commonly used model ecotype Col-0 is highlighted in red and three particularly unresponsive ecotypes that were selected for further experiments are shown in yellow.

Plants exhibit lower leaf temperatures when subjected to low CO_2_ concentrations because these conditions make the stomata open, resulting in increased evaporation. Conversely, plants exhibit higher leaf temperatures when the stomata close under high CO_2_ concentrations. Subtractive thermographs can be used to visualize CO_2_-dependent changes in leaf temperature that represent CO_2_ responsiveness [14]. We used subtractive images to investigate stomatal responsiveness to changes in CO_2_ concentrations in 374 *Arabidopsis* ecotypes, and selected 47 ecotypes for measurements of stomatal conductance; among these were the ecotypes that showed relatively small increases in leaf temperature in response to high CO_2_ concentrations in comparison to the commonly used model ecotype Col-0. Stomatal conductance analysis was performed using a gas exchange system. Fig. 1 shows the relative conductance levels (*g*_*s*_ at 1,000 ppm CO_2_)/(*g*_*s*_ at 0 ppm CO_2_) for the 47 ecotypes. Higher relative conductance levels indicate lower responsiveness to high CO_2_ concentrations. The ecotypes that showed the smallest increases in temperature in response to high CO_2_ concentrations also showed the highest relative conductance levels. These three ecotypes, Ga-0, Chi-1, and Kl-4 (yellow bars in Fig. 1), exhibited particularly low responsiveness to CO_2_. They were investigated in more detail.

### Kl-4, Ga-0, and Chi-1 were impaired in stomatal opening in response to low CO_2_ concentration

**Fig 2:**
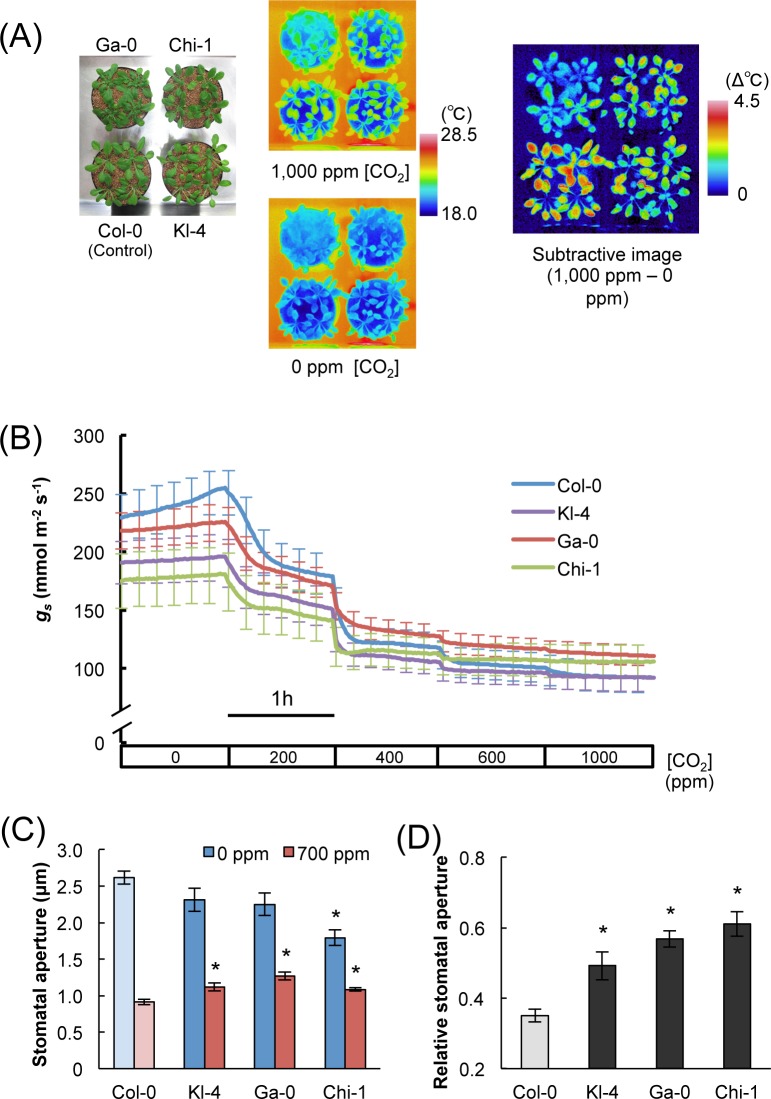
The phenotype of Kl-4, Ga-0, and Chi-1, which exhibit low CO_2_ responsiveness. (A) Thermal imaging of the three selected ecotypes Kl-4, Ga-0, Chi-1, and the commonly used ecotype Col-0. Plants were subjected to 0 ppm CO_2_ for 2 h and then 1,000 ppm CO_2_ for 1 h at 40% RH. The subtractive image on the right shows that the largest temperature changes were exhibited by Col-0. (B) Time courses of stomatal conductance (*g*_*s*_) in response to changes in CO_2_ concentration in Kl-4, Ga-0, Chi-1, and Col-0. Col-0 is more responsive to changes in CO_2_ concentration than Kl-4, Ga-0, Chi-1. (C) Sizes of stomatal apertures at low and high CO_2_ concentrations. Plants were subjected to 0 ppm CO_2_ for 2 h and then transferred to 700 ppm CO_2_ for 1 h at 40% RH with 150 μmol m^-2^ s^-1^ photosynthetically active radiation. (D) The relative changes in stomatal aperture (relative stomatal aperture) were calculated as (stomatal aperture in 0 ppm CO_2_)/(stomatal aperture in 700 ppm CO_2_). Large values represent small responses. Data presented are means ± SE (*n* = 60) of five independent experiments. Significant differences from Col-0 at *p* < 0.05 (Student’s t test) are indicated by asterisks.

**Fig 3:**
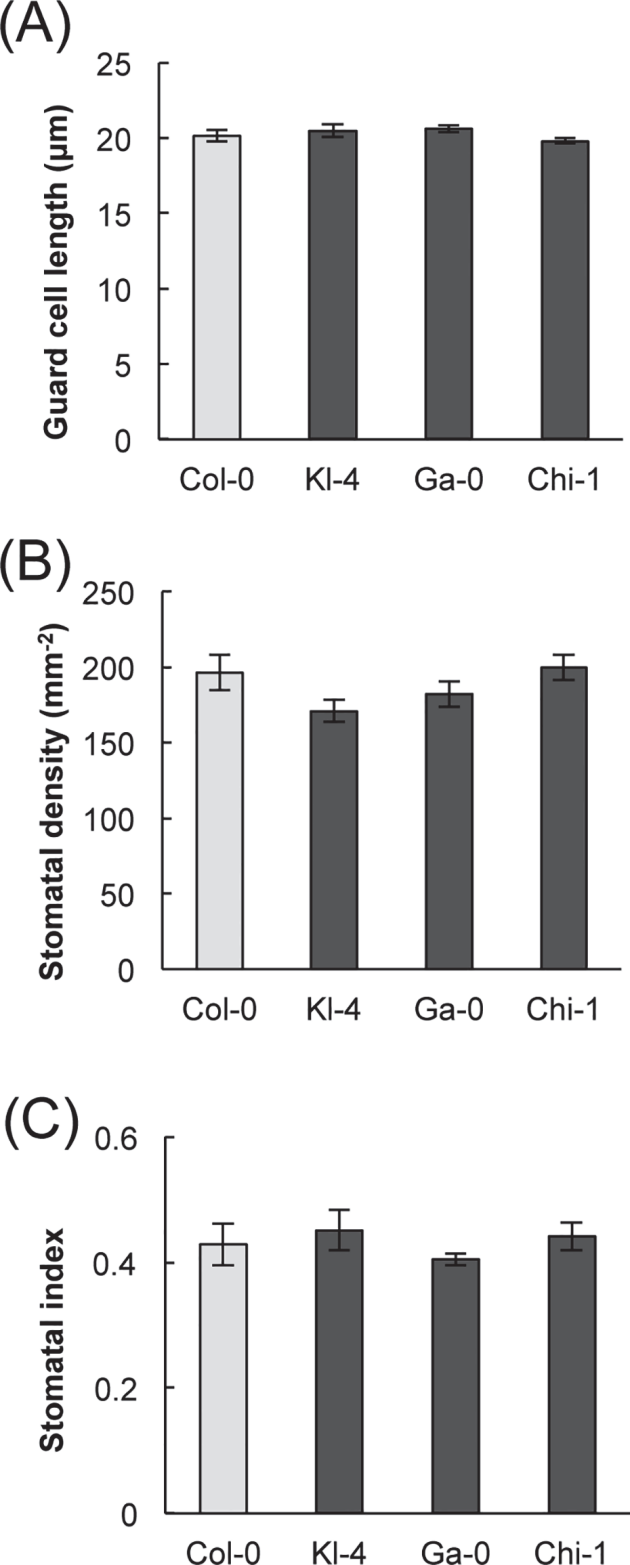
Measures of stomatal size, density, and index in Kl-4, Ga-0, Chi-1, and Col-0. (A) Guard cell lengths, which were used as a measure of stomatal size. (B) Stomatal densities. (C) Stomatal index. Data presented are means ± SE (*n* = 3). No significant differences were observed between the four ecotypes.

We compared the three selected ecotypes and Col-0 in their temperature responses to changes in CO_2_ concentrations. The three selected ecotypes showed relatively small temperature differences, indicating the comparatively low CO_2_ responsiveness of the stomata (Fig. 2A). Next, we measured stomatal conductance in four ecotypes at various CO_2_ concentrations ranging from 0 to 1,000 ppm. The stomatal conductance levels of the three selected ecotypes were lower than those of Col-0 at low CO_2_ concentrations, however, they exhibited similar but slightly higher stomatal conductance levels when compared with Col-0 at high CO_2_ concentrations (Fig. 2B). This indicated that, when compared with Col-0, the three selected ecotypes showed lower responsiveness to CO_2_ in stomatal conductance as well as in leaf temperature. Stomatal conductance is determined predominantly by stomatal size, density, and aperture [23]. We found no differences in stomatal size, index, or density among the four ecotypes (Fig. 3A, B, C). On the other hand, the three selected ecotypes showed significantly smaller changes in stomatal aperture with an increase of CO_2_ concentration from 0 ppm to 700 ppm, when compared with Col-0 (Fig. 2C). This result was consistent with the thermography and stomatal conductance data. The least responsive of the three ecotypes was Chi-1, followed by Ga-0 and Kl-4 (Fig. 2D).

### Kl-4, Ga-0, and Chi-1 showed low responsiveness to light but normal responsiveness to humidity

**Fig 4:**
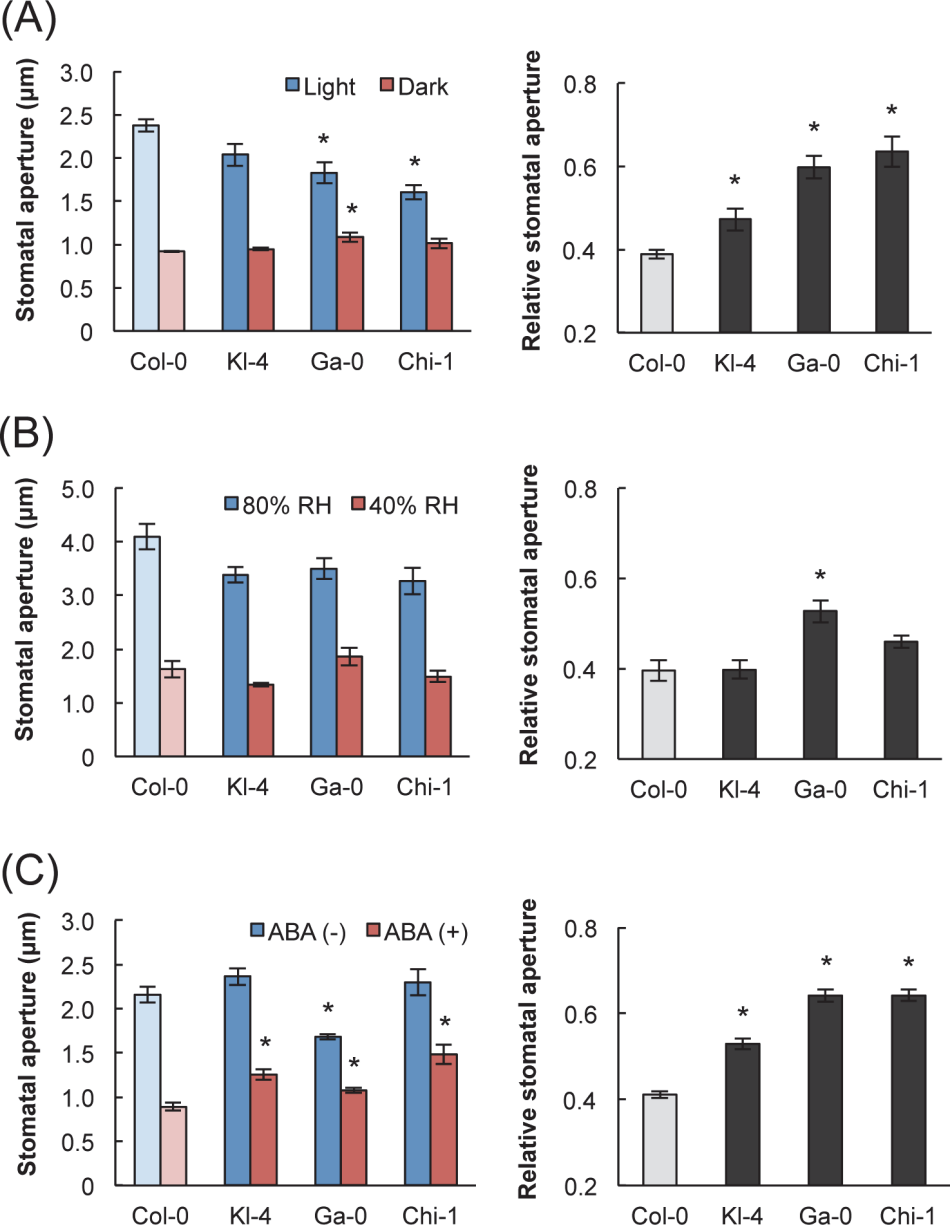
Stomatal response to light, humidity, and ABA in Kl-4, Ga-0, Chi-1, and Col-0. **(A)** The sizes of stomatal apertures under light and dark conditions are shown on the left. The relative changes in stomatal aperture in response to light (relative stomatal aperture) are shown on the right. Plants were subjected to dark conditions for 2 h after illuminated with white light at an intensity of 250 μmol m^-2^ s^-1^ for 3 h. The relative changes were calculated as (stomatal aperture in the light)/(stomatal aperture in the dark). (B) The sizes of stomatal apertures in response to high (80%) and low (40%) RH (left) and humidity-dependent changes of stomatal aperture (right). Plants were kept under 80% RH for 3 h and then transferred to 40% RH for 1 h at 350 ppm CO_2_ and 150 μmol m^-2^ s^-1^ photosynthetically active radiation. The relative changes were calculated as (stomatal aperture in 40% RH)/(stomatal aperture in 80% RH). (C) Influence of ABA on stomatal aperture (left) and ABA-dependent changes in stomatal aperture (right). Epidermal peels were floated on a medium for 1 h, then transferred to the same medium with or without 2 μM ABA, and incubated for a further 2 h. Relative changes were calculated as (stomatal aperture with ABA)/(stomatal aperture without ABA). Data presented are means ± SE (*n* = 60) of five independent experiments. Significant differences from Col-0 at *p* < 0.05 (Student’s t test) are indicated by asterisks.

Next, we investigated how stomatal responsiveness varies with other environmental factors. To measure stomatal responses to light, the plants were first placed in darkness to close the stomata after illuminated for 3h. The three selected ecotypes were impaired in their light-induced stomatal opening response when compared with Col-0 (Fig. 4A). Similarly to their CO_2_ responses of the three ecotypes, Chi-1 was the least responsive to light and Kl-4 was the most responsive. Next, humidity was varied. Plants were transferred from high-humidity (80% RH) to low-humidity conditions (40% RH). In contrast to the responses to CO_2_ and light, the responses to changing humidity did not consistently differ between the three selected ecotypes and Col-0 (Fig. 4B). We also investigated the plants’ responses to ABA, which is involved in stomatal closure under drought conditions. The Kl-4, Ga-0, and Chi-1 showed reduced responsiveness to ABA compared with Col-0, indicating that they are impaired in ABA-induced stomatal closing. This result contrasted with the plants’ responses to humidity (Fig. 4C).

### Kl-4, Ga-0, and Chi-1 showed reduced accumulation of K^+^ and Cl^-^ in the light

**Fig 5:**
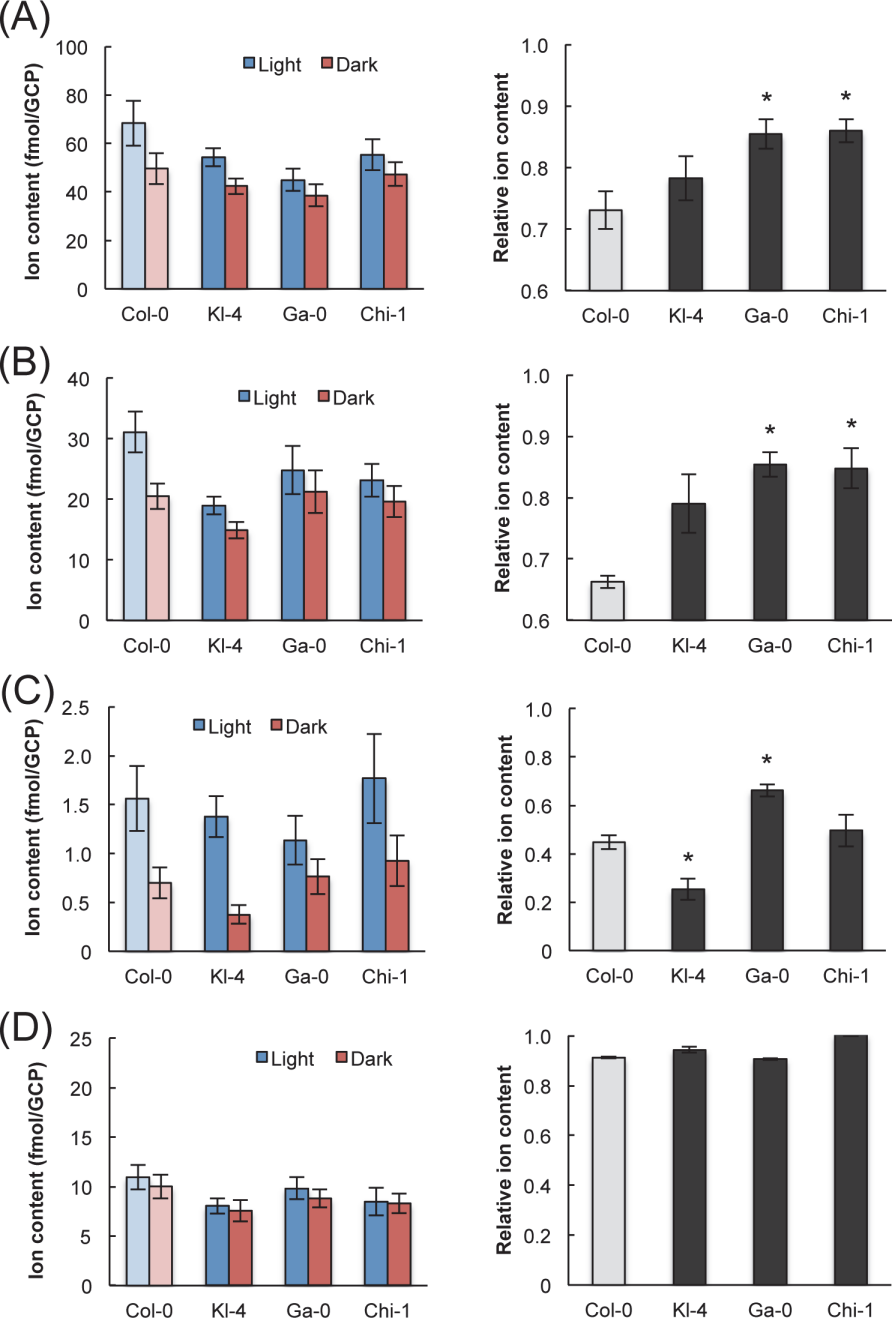
K^+^, Cl^-^, Malate^2-^, and Na^+^ levels in guard cell protoplasts. Ion contents were measured in GCPs from each ecotype under light and dark conditions. The GCPs were incubated with or without white light (70 μmol m^-2^ s^-1^) for 1 h; this amount of light is sufficient to induce GCP swelling and stomatal opening in intact leaves. Relative ion content changes were calculated as (ion content in the dark)/(ion content in the light). Each part of the figure shows ion content data on the left and relative ion contents on the right. Data are for K^+^ (A), Cl^-^ (B), Malate^2-^ (C), and Na^+^ (D). The data presented are means ± SE (*n* = 5–6). Significant differences from Col-0 at *p* < 0.05 (Student’s t test) are indicated by asterisks.

An influx of K^+^ ions triggers stomatal opening by increasing the osmotic pressure within the guard cells. Cl^-^ and Malate^2-^ are the primary anions that counterbalance the influx of K^+^ during stomatal opening [24, 25]. Since the light responsiveness of the three selected ecotypes was low compared with Col-0, we investigated the possibility that the efficiency of ion homeostasis in guard cells may be involved. To measure ion content of guard cells, isolated GCPs were incubated with or without white light. In agreement with the stomatal responses to light, the light-dependent accumulation of K^+^ and Cl^-^ in the three selected ecotypes was less pronounced than in Col-0 (Fig. 5). These results suggest that the light responsiveness may be caused by differences in the regulation of ion transport across the guard cell plasma membrane. Interestingly, the accumulation of Malate^2-^ in GCPs of the three selected ecotypes showed a different pattern from those of K^+^ and Cl^-^, and the regulation of Malate^2-^ content differed among Kl-4, Ga-0, and Chi-1.

## Discussion

**Fig 6:**
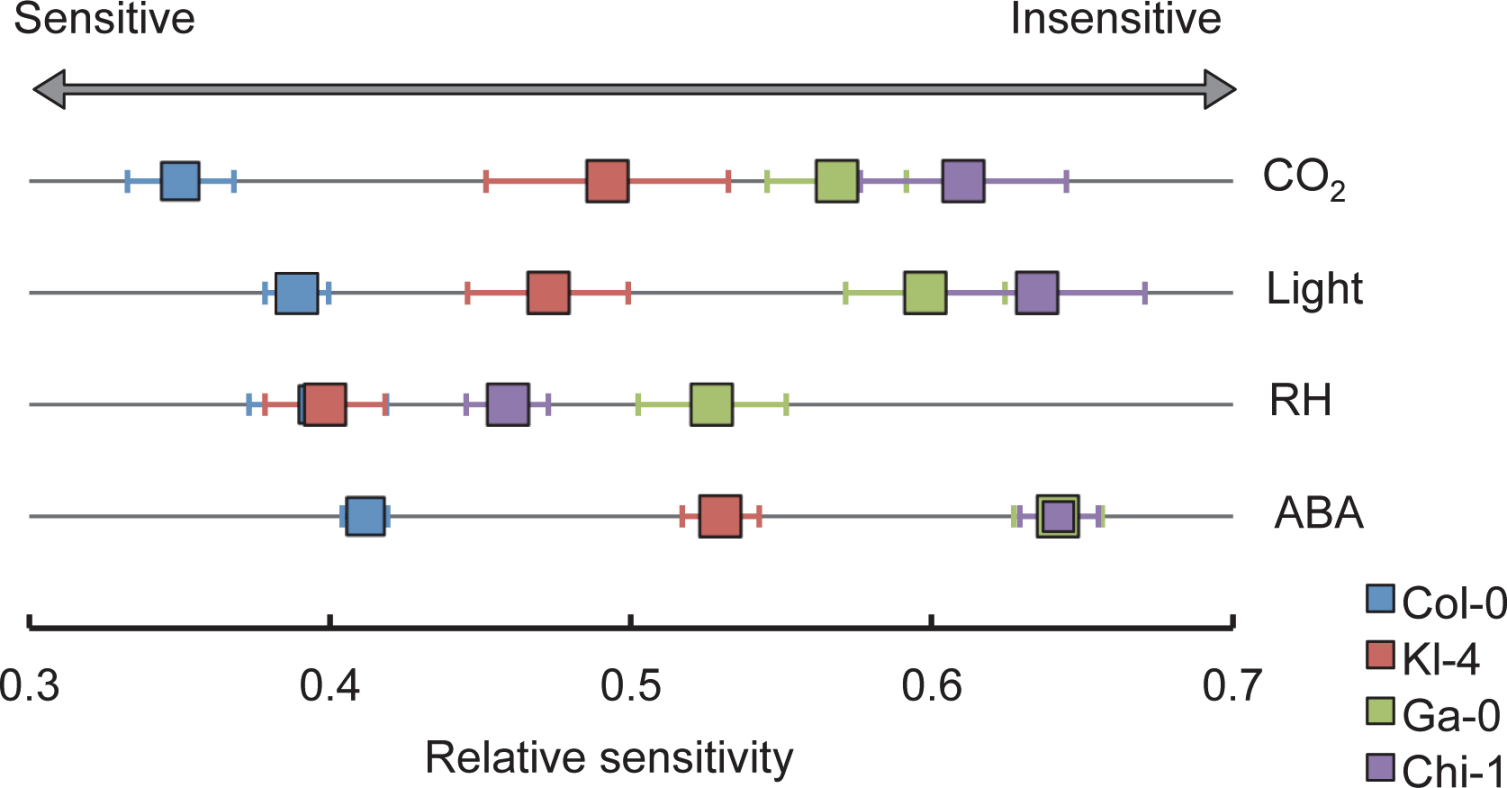
Relative sensitivities to environmental factors among Kl-4, Ga-0, Chi-1, and Col-0. The diagram summarizes the relative sensitivities of Kl-4, Ga-0, Chi-1, and the reference ecotype Col-0 to CO_2_, light, humidity, and ABA. Kl-4 and Col-0 show similar levels of sensitivity to changes in RH. Chi-1 and Ga-0 show similar levels of sensitivity to ABA.

Plant phenotyping technology has become more advanced with the capacity to measure many morphological and physiological traits in each individual [26]. With thermal imaging it is possible to investigate stomatal regulation in large number of plants [4, 18]. In this study, we used thermography to investigate stomatal responses to changes in CO_2_ concentrations in 374 *Arabidopsis* ecotypes. This is a novel approach to the comprehensive investigation of natural variation in stomatal responsiveness. We found large variations in temperature responsiveness to CO_2_ among the ecotypes, indicating variations in stomatal responsiveness to CO_2_. Three ecotypes, Kl-4, Ga-0, and Chi-1, showed particularly low responsiveness to CO_2_ in comparison with the ecotype Col-0, and we investigated the stomatal responses of these ecotypes to other environmental factors. Fig. 6 summarizes the relative sensitivities of Kl-4, Ga-0, Chi-1, and Col-0 to CO_2_, light, relative humidity, and ABA. The ranking of the ecotypes in their sensitivity to light is the same as the ranking for their CO_2_ sensitivity, suggesting that the CO_2_ and light signaling pathways to stomatal opening are coupled. Earlier studies also indicate a link between CO_2_ and light signaling in guard cells. Roelfsema *et al*. (2002) investigated the membrane potentials of guard cells in intact *Vicia faba* plants after exposure to CO_2_ and/or a beam of red light [27]. The authors concluded that guard cells responded to a red light-induced reduction in CO_2_ concentration in the substomatal cavity rather than responding more directly to the red light.

The sensitivities of Kl-4, Ga-0, Chi-1 to changes in humidity show a different pattern than those for CO_2_ and light (Fig. 6). Furthermore, there is a pronounced difference in the sensitivity patterns for humidity and ABA, suggesting that the low-humidity-induced signaling pathway and the ABA-dependent signaling pathway for stomatal closure may be differently affected among the individual ecotypes. This finding appears to conflict with the results of Xie *et al*. (2006), who found disruptions in both RH signal transduction and the ABA-signaling network in the *aba2* and *ost1* mutants [13]. However, another report suggests that ABA signaling and low RH sensing function via distinct pathways. Assmann *et al*. (2000) measured stomatal responses to humidity in ABA-deficient (*aba1*) and ABA-insensitive (*abi1-1* and *abi2-1*) mutants [28]. The ability of the guard cells to sense changes in humidity was not diminished even in plants with dramatically altered ABA levels or ABA-sensing mechanisms. Our observation supports this result.

We found no correlations between stomatal responsiveness to CO_2_ and climate data, growing seasons, or geographical origins of the ecotypes. This was partly expected because the rising atmospheric CO_2_ concentrations are likely to be relatively new phenomena arising from increased industrialization. Such phenomena could not have been major factors influencing the spread of *Arabidopsis* ecotypes. Molecular polymorphisms that correlate with the low CO_2_ responsiveness phenotype could have been maintained in some ecotypes without selection pressure. However, this may not be the case for Ga-0, one of the three ecotypes identified in this study. Previous studies have shown that Ga-0 has reduced sensitivity to O_3_ [29] and is resistant to plantago asiatica mosaic virus [30] and *Fusarium graminearum* [31]. These traits may be related to the weakened stomatal opening trait of Ga-0 because O_3_ uptake and the entry of some pathogens mainly occurs through stomata. For example, the *slac1* mutant, which has constitutively high stomatal conductance, exhibits O_3_-sensitivity because of the restricted stomatal closure under O_3_ exposure [15]. Stomatal closure is induced by pathogen-associated molecular patterns and is a mechanism that can restrict bacterial invasion [32], although many pathogens can force entry through closed stomata.

Further studies are needed to elucidate in detail the mechanisms regulating stomatal responsiveness to different environmental factors. Ion homeostasis data will contribute to the investigation of intracellular functions. Malate^2-^ is the osmoregulatory organic anion responsible for stomatal opening, along with inorganic ions such as K^+^ and Cl^-^. Malate^2-^ and Cl^-^ efflux from guard cells through anion channels and depolarize the cell membrane [23], and this in turn drives K^+^ efflux from guard cells during stomatal closure [24]. While the regulation of homeostasis is central to the control of stomatal response, it remains unclear how different environmental conditions alter the activities of the K^+^, Cl^-^, and Malate^2-^ channels in the three selected ecotypes. Our study will advance our understanding of the mechanisms by which different environments can alter the phenotypes of individual genotypes, and how genotypes can differ in response to the same environment. Taken together, our results underline the high potential of comparative studies of natural ecotypes for improving our understanding of environmental information processing in plants.

## Supporting Information

S1 Table Ecotypes used in thermal imaging, their subtractive leaf temperatures, and the latitudes and longitudes of their collection sites. The relative subtractive leaf temperature was calculated as (change in leaf temperature of the ecotypes)/(change in leaf temperature of Col-0). The change in leaf temperature is the average of measurements from three different leaves in the subtractive images. Plants grown in a growth chamber were transferred to a growth cabinet (constant white light of 100 μmol m^-2^ s^-1^ at 22°C, 60% RH) equipped with an automatic CO_2_ control unit (FR-SP, Koito). Plants were subjected to 0 ppm CO_2_ for 1.5 h and then 700 ppm CO_2_ for 1 h. Thermal images were captured under each CO_2_ concentration using a thermography camera (TVS-8500, NEC/Avio). CS numbers refer to ABRC stock numbers. The latitude and longitude information was gathered from the Arabidopsis Information Resource web site.
(XLSX)
